# Whole-Genome Expression Analysis in the Third Instar Larval Midgut of *Drosophila melanogaster*

**DOI:** 10.1534/g3.114.013870

**Published:** 2014-09-05

**Authors:** Thomas W. R. Harrop, Stephen L. Pearce, Phillip J. Daborn, Philip Batterham

**Affiliations:** Department of Genetics, Bio21 Molecular Science and Biotechnology Institute, The University of Melbourne, Victoria, Australia

**Keywords:** metabolism, xenobiotic, insecticide, P450, GST

## Abstract

Survival of insects on a substrate containing toxic substances such as plant secondary metabolites or insecticides is dependent on the metabolism or excretion of those xenobiotics. The primary sites of xenobiotic metabolism are the midgut, Malpighian tubules, and fat body. In general, gene expression in these organs is reported for the entire tissue by online databases, but several studies have shown that gene expression within the midgut is compartmentalized. Here, RNA sequencing is used to investigate whole-genome expression in subsections of third instar larval midguts of *Drosophila melanogaster*. The data support functional diversification in subsections of the midgut. Analysis of the expression of gene families that are implicated in the metabolism of xenobiotics suggests that metabolism may not be uniform along the midgut. These data provide a starting point for investigating gene expression and xenobiotic metabolism and other functions of the larval midgut.

In insects, the metabolism of xenobiotics is vital to allow survival in environments that contain toxic substances such as secondary plant metabolites or insecticides, which may have evolved or been developed to control insect feeding or reproduction. The insect midgut is an unprotected interface with the environment (Li *et al.* 2008), and several lines of evidence indicate that along with the Malpighian tubules and fat body, it is a primary site of xenobiotic metabolism. For example, the expression of gene families involved in insecticide metabolism is enriched in the midgut of *Drosophila melanogaster* (Li *et al.* 2008), and transgenic overexpression of various members of these gene families in the midgut, Malpighian tubules, and fat bodies confers resistance to insecticides (Chung *et al.* 2007; Daborn *et al.* 2007, 2012), and in honeybees, the products of at least two separate metabolic pathways were detected in the midgut after feeding them with radiolabeled imidacloprid, a neonicotinoid insecticide (Suchail *et al.* 2004).

Gene expression in databases such as FlyAtlas and modENCODE is reported for intact midguts (Chintapalli *et al.* 2007; Celniker *et al.* 2009), but there are differences in gene expression, cell type, morphology, and lumen acidity in different subsections of the midgut. Based on the pH in the lumen, the midguts of insects can be separated into at least three compartments (Terra and Ferreira 1994). The expression of antimicrobial peptides suggests that the anterior midgut may be involved in immune function (Tzou *et al.* 2000). The middle midgut, which is often acidic (Terra and Ferreira 1994), contains three subsections, the copper cell, large flat cell, and iron cell regions. Two cell types have been described in the copper cell region: Copper cells, which acidify the gut, and interstitial cells, which have an unknown function (Dubreuil 2004). The large flat cell region may be involved in food digestion and the iron cell region may neutralize the lumen, as the posterior midgut is less acidic than the middle midgut (Filshie *et al.* 1971).

Compartmentalized gene expression indicates that the midgut is an even more complex tissue than its morphology suggests. Thirteen distinct compartments of expression were identified by enhancer trap analysis of Drosophila larvae, with M1–5 comprising the anterior midgut, M6–8 the middle midgut, and M9–13 the posterior midgut (Murakami *et al.* 1994), and recent enhancer- or protein-trap analyses performed on 6- to 8-day-old adult flies largely recapitulate the compartments of expression reported in larvae (Murakami *et al.* 1994; Marianes and Spradling 2013; Buchon *et al.* 2013). In adult midguts, 10 different cell morphologies along the anteroposterior axis were identified (Marianes and Spradling 2013), corresponding to five major divisions containing 13 subregions, which were indicated by anatomical, histological, and gene expression analysis (Marianes and Spradling 2013; Buchon *et al.* 2013). RNA sequencing within subsections revealed expression of genes encoding proteins that may be involved in digestion in the large flat cell and iron cell regions, and functional annotation analysis of these results was used to suggest a general scheme for midgut function in adults (Marianes and Spradling 2013).

The sections of the midgut that are important for metabolism of xenobiotics in larvae have not been identified, and xenobiotic metabolism in insects is not well understood, with much of the available information derived from studies investigating *in vitro* metabolism of insecticides. Although expression of metabolic genes in the nervous system can result in insecticide resistance (Korytko and Scott 1998; Zhu *et al.* 2010), metabolism of imidacloprid occurs in the midgut of *D. melanogaster* larvae and the metabolites are rapidly excreted (Hoi *et al.* 2014). Detoxification often is described with terms that follow the scheme of drug disposition in mammals, whereby ‘Phase I’ enzymes encoded by the cytochrome P450 (P450) and carboxylesterase gene families oxidize, hydrolyze, or reduce xenobiotics, and the products of these reactions are conjugated to certain substrates by ‘Phase II’ enzymes encoded by the glutathione *S*-transferase (GST) and UDP-glycosyltransferase (UGT) families to allow them to be excreted (reviewed in Kadlubar and Kadlubar 2009). However, the metabolism of xenobiotics *in vivo* in insects is likely to be far more complex. For example, the *D. melanogaster* P450 enzyme CYP6G1 can metabolize the neocotinoid insecticide imidacloprid to several products, including those that may be more toxic than the original compound, when expressed in tobacco cell culture (Joußen *et al.* 2008). Some other cytochrome P450 enzymes also have the capability to perform reactions that increase the toxicity of insecticides (Dunkov *et al.* 1997; Guzov *et al.* 1998; Sabourault *et al.* 2001; Honda *et al.* 2006; Hoi *et al.* 2014), suggesting that describing the members of these gene families as “detoxification genes” may not accurately reflect their functions.

In this study, transcriptome analysis was used to investigate gene expression relevant to xenobiotic metabolism in *D. melanogaster*, which is an established model for studies of the genetics of resistance to xenobiotics, in particular insecticides (Perry *et al.* 2011). Midguts from feeding third instar larvae were dissected into eight subsections following the regions of gene expression determined by enhancer trap analysis in larvae (Murakami *et al.* 1994), and high-throughput mRNA sequencing was used to estimate gene expression in each subsection. Consistent with previous reports (Murakami *et al.* 1994; Buchon *et al.* 2013; Marianes and Spradling 2013), expression in the midgut is compartmentalized. The expression of families of genes implicated in xenobiotic metabolism was detected in a variety of patterns, suggesting that metabolism may occur along the length of the midgut, although polarization of expression of several metabolic gene families was observed, such that they are generally more lowly expressed in the middle midgut. In addition, the analysis identified several common patterns of expression among highly expressed, variable genes. These results support differentiated function between subsections, and the data will be a useful resource for further investigation of xenobiotic metabolism and other functions of the larval midgut.

## Materials and Methods

### Dissections and RNA isolation

To guide dissection, a *D*. *melanogaster* line that expresses *GAL4* in M2, M4, M6 and M9 and M12–13 was produced by *P*-element–mediated transformation with a recombinant *P*{CaSpEr-*GAL4*} plasmid containing the promoter of *Cyp4d2* upstream of the *GAL4* gene (data not shown). Flies homozygous for this transgene were crossed with *w*^1118^ ; *P*{UAS-*GFP*.nls}14 flies (Bloomington stock number 4775). This made it possible to dissect eight subsections of the midgut in the green fluorescent protein (*GFP*)-expressing larval progeny, corresponding to the M1, M2, M3–5, M6, M7–8, M9, M10–11, and M12–13 subsections identified by enhancer-trap analysis (Murakami *et al.* 1994). Larvae were raised on standard cornmeal-dextrose medium with 12 mL·L^−1^ of 10% w/v methylparaben (Lewis 1960). Midguts were dissected at the feeding stage of third instar larval development under an SZX12 stereomicroscope (Olympus) in batches of five from a minimum of 200 larvae and transferred to RNA*later* solution (QIAGEN). Total RNA was extracted using the RNeasy Lipid Tissue Mini Kit (QIAGEN) and RNA integrity was assayed on a BioAnalyzer (Agilent). Unstranded library preparation and sequencing on the Illumina GAIIx platform were carried out by Macrogen (Seoul, Korea).

### Sequencing and analysis

Sequencing produced 35–38 million 38-bp single-end reads for each sample, except M6. RNA from the M6 section was more prone to degradation than RNA from other sections (Marianes and Spradling 2013), and only 17 million reads were produced for this section. Low quality reads (Q < 3) were removed with PRINSEQ 0.20.3 (Schmieder and Edwards 2011), and the remaining reads were mapped to the annotated *D. melanogaster* genome (FlyBase release 5.47) using Bowtie 2.0.2 and TopHat 2.0.6 (Kim *et al.* 2013). Fragments per kilobase of exon per million fragments mapped (FPKM) quantification and testing for differential expression were performed using Cuffdiff 2.0.2 with options for upper-quartile normalization (−N), multiread correction (−u), fragment bias correction (−b), and timeseries (−T) enabled, using the “blind” dispersion method, which constructs a conservative model in the case of nonreplicated samples (Trapnell *et al.* 2013). Raw and processed data files, including tables of FPKM estimates output by cuffdiff that were used to generate heatmaps, are available from the National Center for Biotechnology Gene Expression Omnibus under the accession number GSE32329. Analysis and visualization of the processed RNAseq data and generation of heat maps were performed using the R statistical computing environment (R Foundation for Statistical Computing, Vienna, Austria). Fuzzy C-means clustering was performed with the Mfuzz package (Kumar and Futschik 2007).

## Results and Discussion

### Patterns of gene expression and regulation in the midgut

**Figure 1:**
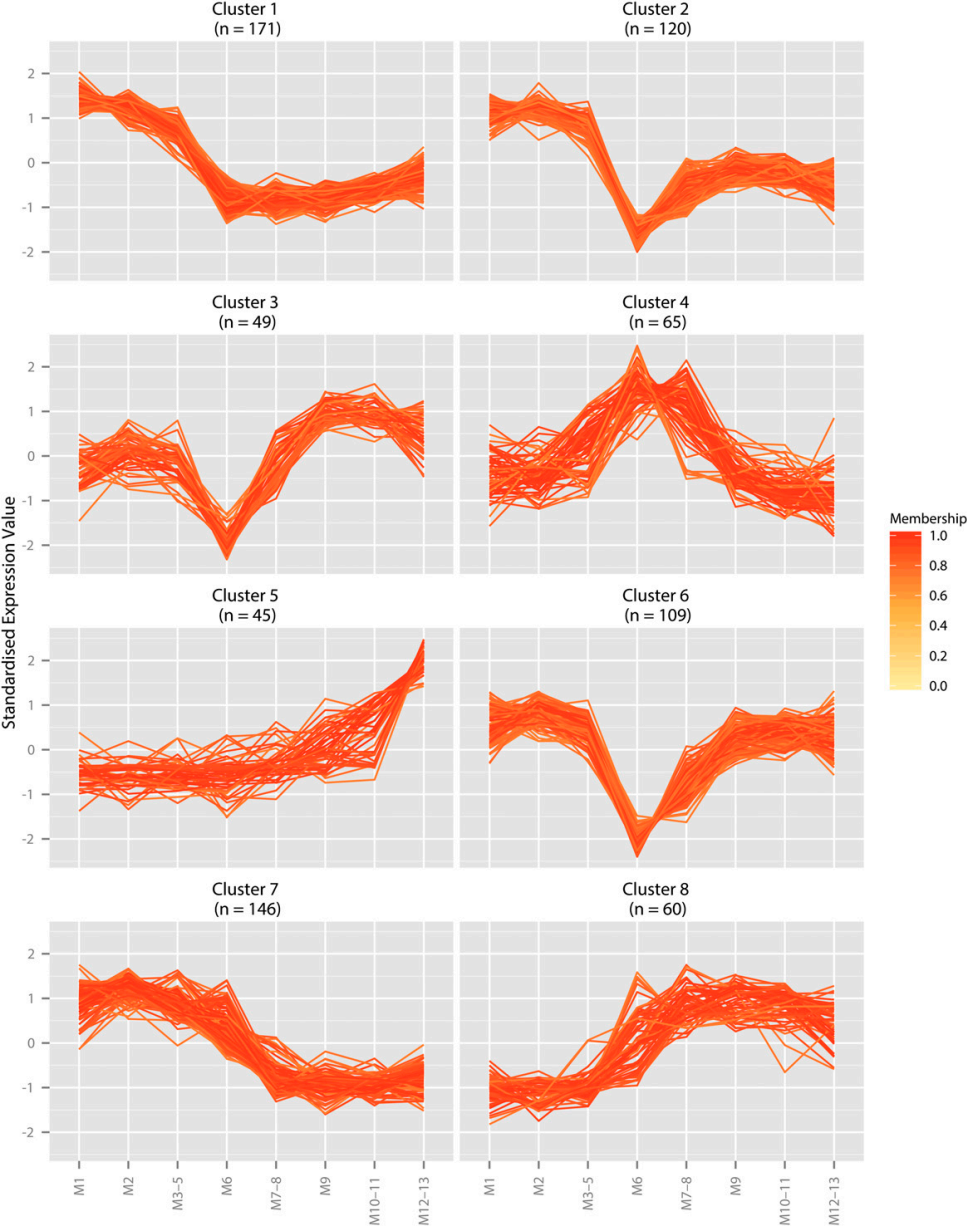
Fuzzy C-means clustering of genes with variable expression in the larval midgut. Eight clusters were formed, including three separate clusters with markedly decreased expression in M6 (clusters 2, 3, and 6) and one cluster with specific expression in the middle midgut (cluster 4). The clustering supports previous findings that gene expression within the adult midgut is variable, suggesting that the subsections are also functionally differentiated in larvae. Scale bar: fractional membership of each gene to the cluster.

**Figure 2:**
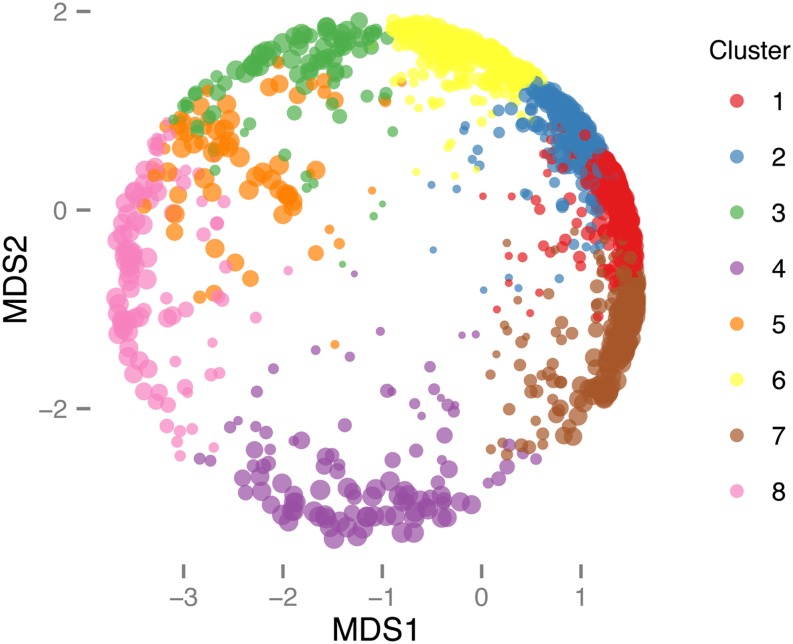
Multidimensional scaling (MDS) of standardized, log-transformed FPKM values of clustered genes, scaled by α, which is the gene’s membership value for the cluster to which it was assigned, larger points having α values closer to 1. The clusters are supported by the MDS analysis. FPKM, Fragments per kilobase of exon per million fragments mapped.

To investigate the patterns of gene expression in the subsections of the midgut, fuzzy C-means clustering was performed on standardized, log-transformed FPKM values from a subset of highly expressed genes with variable expression. To construct this gene set, genes with FPKM > 5 in any section or mean FPKM > 3 across all sections (6315 genes) were ranked by variance across all sections, and the top 20% were retained for fuzzy C-means analysis (1263 genes). The retained genes define 8 clusters with differing patterns of expression along the length of the midgut (Figure 1). Multidimensional scaling of a distance matrix of standardized, log-transformed FPKM values (*i.e.* principal component analysis) supports the separation of the expression clusters (Figure 2). The expression of genes in some clusters reflects the divisions between the anterior, middle, and posterior midgut, corresponding to known physiological or morphological distinctions, and the differential patterns of gene expression indicate that the subsections of the third instar larval midgut may have differences in function. For example, genes in cluster 1 are enriched in the anterior midgut and more lowly expressed in the middle and posterior midgut, whereas cluster 8 contains genes expressed in the posterior and middle midgut. Cluster 5 may reveal differences of expression in morphologically indistinguishable compartments of the larval midgut, as it contains genes specifically expressed in M12–13 but not the other sections of the anterior midgut.

The “core” genes in each cluster, which have a cluster membership value (α) of at least 0.7, were manually examined for the presence of members of gene families that may be involved in xenobiotic metabolism (Supporting Information, File S1). Clusters 2, 3, and 6, which have genes that are expressed in regions of the midgut excluding M6–8, contained P450s, GSTs, carboxylesterase, and UGTs, which supports polarization of xenobiotic metabolism in the midgut (*Xenobiotic metabolism in the midgut*). However, *α-Est3*, *Cyp6a13*, and *GstE10* are all found in cluster 4, containing genes expressed specifically in the middle midgut (M6–8), suggesting that the polarization of the activity of these gene families is not absolute.

These data corroborate previous reports where compartment-specific expression in the midgut was studied using enhancer-trap analysis (Murakami *et al.* 1994; Marianes and Spradling 2013), but the transcription factors that coordinate differential expression in the midgut are unknown. A manually curated list of transcription factor genes in *D. melanogaster* is available (Hammonds *et al.* 2013), and four of these were found in the lists of core genes. Cluster 2, containing genes with high expression in the anterior midgut, lower expression in the anterior midgut and little to no expression in the middle midgut, includes the ubiquitously expressed basic leucine-zipper transcription factor *X box binding protein-1* (FBgn0021872; Liou *et al.* 1990; Souid *et al.* 2007). The zinc-finger protein *sugarbabe* (FBgn0033782), which may regulate insulin-like peptide production in insulin producing cells and is activated by sugar ingestion (Zinke *et al.* 2002; Varghese *et al.* 2010), was found in cluster 6, which contains genes widely expressed in the midgut except M6–8. Cluster 7 (high anterior expression transitioning to lower expression in the middle midgut) contains a basic leucine-zipper transcription factor involved in regulation of ecdysone triggering hormone, *cryptocephal* (FBgn0000370; Gauthier *et al.* 2012). *Xrp1* (FBgn0261113), which induces upstream factors involved in axis formation during antenna development (Tsurui-Nishimura *et al.* 2013), was found in cluster 8 (low anterior expression transitioning to higher expression in the middle midgut). No evidence is available for the involvement of these genes in xenobiotic metabolism and further investigation will be necessary to determine if they have a role in regulation in the subsections where they are expressed.

### Xenobiotic metabolism in the midgut

**Figure 3:**
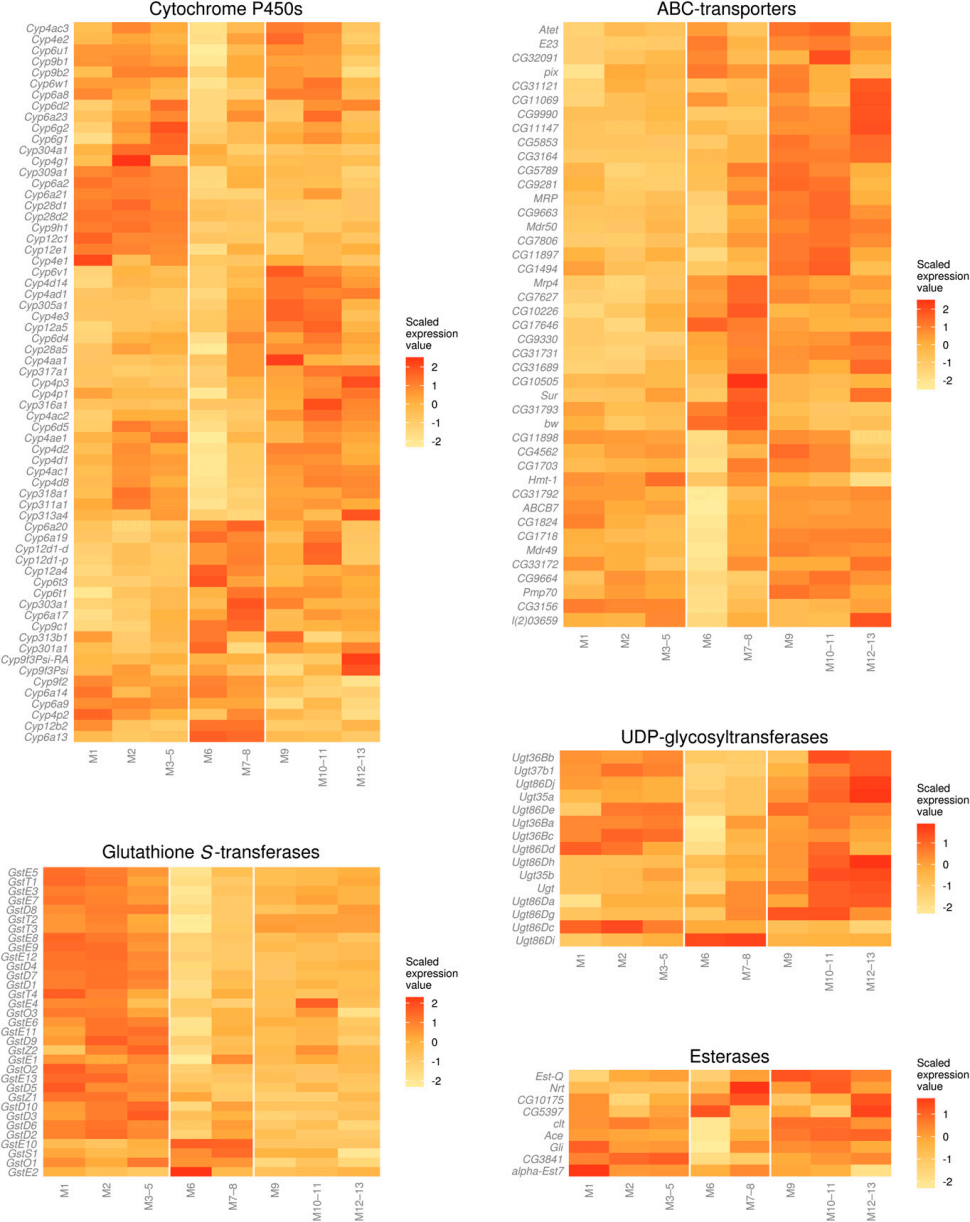
Expression of metabolic gene families and ABC transporters in the midgut. Expression values are log_10_(FPKM + 1), scaled by each gene’s total expression across the 8 subsections. Unscaled FPKM values for these genes are provided in File S2. Genes with low or undetectable expression (sum of FPKM values less than 1) were excluded from the analysis. In general, members of these gene families are not enriched in M6, but there are exceptions, and other patterns of expression within the families were observed (*Xenobiotic metabolism in the midgut*). FPKM, Fragments per kilobase of exon per million fragments mapped.

Genes that are involved in xenobiotic metabolism are often encoded by gene families, including P450s, carboxylesterases, GSTs, and UGTs (Li *et al.* 2007; Perry *et al.* 2011), which are widely expressed in the midgut of *D. melanogaster* and the tobacco hornworm *Manduca sexta* (Li *et al.* 2008; Pauchet *et al.* 2010), although their members are involved in a broad range of biological processes apart from metabolism (Oakeshott *et al.* 2005; Chung *et al.* 2009; Ranson and Hemingway 2005; Feyereisen 2012). Heat maps of expression of these families in subsections of the larval midgut show that their members are extensively expressed in the midgut subsections, with some notable patterns within and between families (Figure 3).

Across all of the gene families, expression was generally lower in the M6 (copper cell) subsection. This finding suggests that M6 is not a prominent site of xenobiotic metabolism, which is consistent with its proposed function in acidification of the lumen (Dubreuil 2004). Approximately two-thirds of P450s are not enriched in M6, including a large group of P450 genes that are expressed in all or most of the midgut but absent or lowly expressed in M6–8, *e.g. Cyp6g1*, which metabolizes DDT and imidacloprid and is involved in resistance to several insecticides in the field (Daborn *et al.* 2002; Joußen *et al.* 2008; Schmidt *et al.* 2010; Hoi *et al.* 2014), and *Cyp6a8* and *Cyp6w1*, which are overexpressed in some resistant strains (Maitra *et al.* 1996; Brandt *et al.* 2002; Le Goff *et al.* 2003). Another P450 linked to resistance by overexpression, *Cyp6a2* (Brun *et al*. 1996; Pedra *et al.* 2004), is specifically enriched in the anterior compartments. There is also a group of P450s that are strongly expressed in M6–8, such as *Cyp12a4*, which confers resistance to the insect growth regulator, lufenuron, when overexpressed in natural populations or by transgenic manipulation (Bogwitz *et al.* 2005), and another resistance-associated P450, Cyp12d1 (Brandt *et al.* 2002; Le Goff *et al.* 2003; Daborn *et al.* 2007), although both of these genes also have prominent expression in M10–11. There are also several P450s that are enriched in the posterior compartments (*e.g. Cyp305a1*, *Cyp4e3*). These observations are consistent with P450-based insecticide metabolism occurring along the length of the third instar larval midgut, with a possible decrease in metabolism in M6–8.

As is the case for P450s, a decrease in the expression of GSTs is apparent in M6–8. Two notable exceptions to this are *GstE10* and *GstS1*, which have their greatest expression in M6 and M7–8, respectively. *GstD1*, which encodes a GST that has metabolic activity toward DDT *in vitro* and has a radical amino acid substitution that seems to be adaptive in some Drosophila species (Tang and Tu 1994; Low *et al.* 2007), is highly expressed throughout the midgut. GSTs are clearly more prevalent in the anterior than posterior midgut, and this pattern makes their expression appear more uniform than the expression of P450 genes.

UGTs catalyze the conjugation of glucuronic acid to their substrate, making it more polar and enabling its excretion. They are not widely studied in xenobiotic metabolism in insects, although *BmUGT1* in *Bombyx mori* encodes an enzyme that was able to conjugate several xenobiotics after heterogeneous expression in cell culture (Luque *et al.* 2002). UGTs are evenly distributed in the midgut, although there may be common enrichment in the M10–11 and M12–13 subsections, and decreased enrichment in M6–8 is apparent.

Only 9 of 22 carboxylesterase genes identified in the RNA sequencing data were detected in the midgut (Σ FPKM ≥ 1), and the expression of those genes that are enriched appears evenly distributed, although 6 of those 9 are less enriched in M6. The expression of *α-Est7* is detected in all sections of the midgut, with especially high expression in M1. *α-Est7* is not implicated in resistance in the field in Drosophila, but ubiquitous, transgenic overexpression provides moderate resistance to diazinon (Birner-Gruenberger *et al.* 2012), and it is the ortholog of the gene encoding the *Lucilia cuprina* carboxylesterase E3, variants of which found in resistant strains have significant hydrolase activity toward organophosphate insecticides (Devonshire *et al.* 2003).

ABC transporters act in metabolism by actively transporting xenobiotics and/or their metabolites into or out of tissues. In theory, efflux could either inhibit or enhance the effect of a xenobiotic. Pumping an insecticide or its metabolite into the lumen of the gut may have a protective effect by allowing its excretion from the insect. Conversely, pumping the insecticide or its metabolite into the hemolymph may facilitate transport to other metabolic tissues or the site of action (*e.g.* the brain). ABC transporters have been implicated in insecticide transport and resistance in arthropods, often because of changes in expression level (reviewed in Dermauw and Van Leeuwen 2014). These genes appear to be more highly expressed in the posterior midgut, also with lower expression in M6. Expression of the *Mdr49* gene, which is implicated in resistance to the plant secondary metabolite colchicine (Wu *et al.* 1991), follows this pattern.

The larvae used in these experiments were raised on normal media, without the addition of insecticide or other xenobiotics, and it is possible that the expression of some genes involved in metabolism is induced by xenobiotics, which would therefore not necessarily be detected under these conditions. Transcriptional response to exposure may be an important distinction between the metabolism of natural xenobiotics and of anthropogenic insecticides—for example, P450s and GSTs can be induced by brief exposure to a high concentration of the natural plant xenobiotic, caffeine but not by several insecticides under the same conditions (Willoughby *et al.* 2006). Nevertheless, many of the genes implicated in insecticide resistance in *Drosophila* were highly expressed in sections of the midgut. A putative mediator of xenobiotic responses, transcription factor Cap ’n’ collar isoform-C, is involved in the regulation of P450s, GSTs, UGTs, and transmembrane transporters in response to phenobarbital (Misra *et al.* 2011). This isoform (FlyBase reference FBtr0306748) was detected in the larval midgut transcriptome dataset, with predominant expression in the middle and posterior midgut (M6–13), and the other isoforms of the *cnc* gene generally follow this expression pattern (data not shown). This expression pattern may be significantly altered by induction with phenobarbital, but because many of the aforementioned genes are expressed in additional subsections, it seems that Cnc is not the only factor involved in constitutive expression of genes involved in xenobiotic metabolism.

### Dataset validation

**Figure 4:**
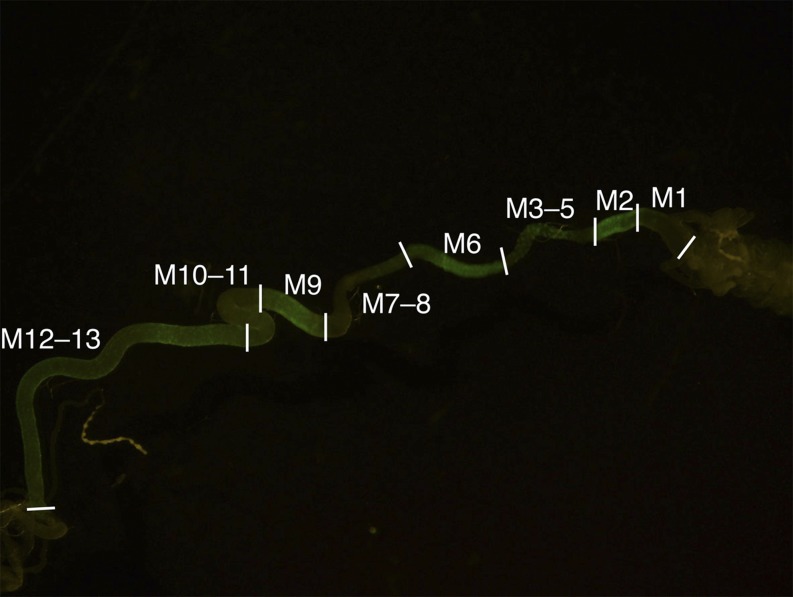
Typical expression in midguts dissected from larvae producing GFP under the control of the *Cyp4d2* promoter. GFP was expressed in M2, M4, M6, M9, M12, and M13, allowing the sections indicated to be harvested for sequencing. GFP, green fluorescent protein.

**Figure 5:**
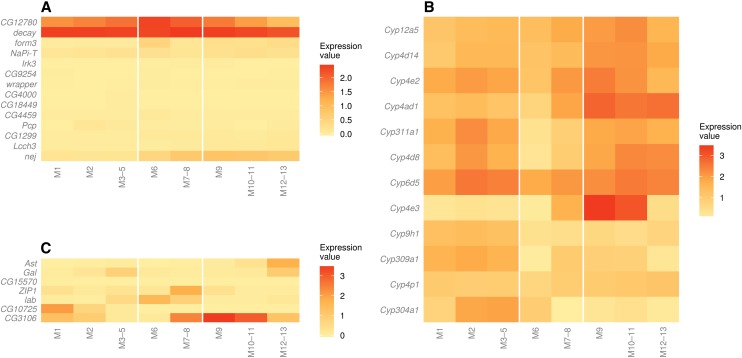
Comparison of the transcriptome data set with three other data sets. Heat maps were produced from the larval midgut dataset for the following groups of genes: (A) genes with restricted expression patterns, chosen from FlyAtlas (Chintapalli *et al.* 2007), (B) 12 P450 genes that were found by RNA *in situ* hybridization to be expressed in subsections of the midgut (Chung *et al.* 2009), and (C) genes used to exclude cross-contamination in RNA from subsections of the adult midgut (Marianes and Spradling 2013). The expression patterns of these genes indicate that the midgut subsections were adequately dissected. Expression values are unscaled log_10_(FPKM + 1).

Expression of GFP in subsections of the midgut, driven with the promoter of the *Cyp4d2* gene, was used to guide dissection of midguts for sequencing (Figure 4). As an initial validation step, the RNA sequencing results were compared with FlyAtlas expression data (Chintapalli *et al.* 2007). For each of eight tissues—the central nervous system, trachea, fat body, hindgut, salivary gland, Malpighian tubule, carcass, and midgut—two genes that are reported by FlyAtlas to be highly and specifically expressed in that tissue were chosen. The expression of these 16 genes was examined in the midgut transcriptome dataset. Only the two genes that are reported by FlyAtlas to be expressed in the midgut were enriched in the midgut transcriptome (Figure 5A). This finding suggests that the midguts were cleanly dissected without contamination with RNA from other tissues.

A previous report presented expression of cytochrome P450 genes in *D. melanogaster* based on RNA *in situ* hybridization, including 12 genes expressed in subsections of the midgut (Chung *et al.* 2009). To determine whether the expression patterns determined by RNA sequencing correspond to the RNA *in situ* hybridization patterns, the expression data for those genes were examined in the midgut transcriptome (Figure 5B). Of the 12 genes compared between the two studies, only two were not enriched in the tissues identified by RNA *in situ* hybridization (*Cyp4d8* and *Cyp311a1*), whereas 10 were (*Cyp4e9*, *Cyp304a1*, *Cyp6d5*, *Cyp309a1*, *Cyp4e2*, *Cyp9h1*, *Cyp4ad1*, *Cyp12a5*, *Cyp4d14*, and *Cyp4p1*). However, transcripts from almost all of the genes in the latter group were enriched in additional tissues not detected by *in situ* hybridization. The RNA sequencing results are therefore supported by the RNA *in situ* results reported by Chung *et al.* (2009), but the differences in the expression patterns suggest that care must be exercised when inferring precise expression patterns from either of the two data sets. Although it is possible that gene expression is different between the transgenic strain used for RNA sequencing and the *y* ; *cn bw sp* strain used for *in situ* hybridization, there are several other possible explanations for the observed differences. For example, the results from RNA sequencing are likely to be more sensitive compared to visualization by *in situ* hybridization, thus allowing detection of expression in compartments missed by the latter technique. Alternatively, imperfect dissection of midgut subsections during sample collection might lead to some amount of RNA from neighboring subsections being detected in the wrong sample. It is also theoretically possible that the expression of GAL4 or GFP altered the expression of some genes in the midgut, but it was not technically feasible to determine if such an effect was present for this data set.

To investigate these differences further, the expression of the seven genes used by Marianes and Spradling (2013) to exclude cross-contamination from imprecise dissection of subsection of the adult midgut was examined in the third instar larval midgut subsections. The enrichment of these genes in subsections of the third instar midgut corresponds closely to the tissues in which enrichment was observed in the adult transcriptome (Figure 5C). This finding suggests that the subsections of the third instar midguts were dissected adequately and the expression patterns estimated from the RNA-sequencing data are likely to be accurate. However, because biological replication was not performed for the midgut sectioning, stochastic variation may result in an incorrect level of expression being reported for any of the genes in any of the sections, and therefore using this RNA sequencing dataset as evidence of a precise level of expression of a particular gene within a certain subsection of the midgut should be avoided, especially for transcripts whose expression varies only slightly from the baseline.

## Conclusion

These results support previous reports that gene expression in the midgut is compartmentalized and indicate that the subsections of the midgut may have important differences in function. The variable expression of genes implicated in xenobiotic metabolism in the midgut, at the level of individual genes and gene families, suggests that metabolism of xenobiotics is not uniform throughout the third instar *D. melanogaster* midgut but rather a complex process that may occur in one or more subsections, perhaps with different steps in metabolism carried out in different regions of the gut. The results of this study provide a starting point for further investigation of xenobiotic metabolism in the midgut, which may be important in understanding and managing metabolic resistance to insecticides.

## Supplementary Material

Supporting Information
