# Supplementary material for: Whole-Genome Expression Analysis in the Third Instar Larval Midgut of Drosophila melanogaster
Source: G3 (Bethesda). 2014 Sep 5;4(11):2197–205. doi: 10.1534/g3.114.013870 (PMC4232545; doi:10.1534/g3.114.013870)
Supplement: Supporting Information [file supp_4_11_2197__index.html]

Whole-Genome Expression Analysis in the Third Instar Larval Midgut of Drosophila melanogaster — Supporting Information 

# Whole-Genome Expression Analysis in the Third Instar Larval Midgut of *Drosophila melanogaster*

## Supporting Information for Harrop *et al.*, 2014

**Files in this Data Supplement:**

- File S1 - Core genes from each cluster (in separate sheets), which have cluster membership value (α) of at least 0.7, corresponding to the genes that were used to produce Figure 1. Columns: FlyBase ID, FlyBase short name (gene.name), FlyBase description, cluster membership (α), FPKM values for the midgut subsections. (.xlsx, 107 KB)
- File S2 - Raw (unscaled) FPKM values for members of the metabolic gene families (in separate sheets), including FlyBase identifier and gene name. (.xlsx, 31 KB)
